# Striatal interneuron microcircuits gate reinforcement to stabilize adaptive choice

**DOI:** 10.64898/2026.09.21.753300

**Published:** 2026-09-22

**Authors:** Evan A. Iliakis, Jonibek M. Muhsinov, Alexandra N. Ramirez, Francesco Rinaldi, Jamie Galanaugh, Saurabh Pandey, Iván Linares-García, Aaron R. Tachau, Eric Z. Song, Luigim Vargas, Kyuhyun Choi, Joel T. Woolley, Sarah M. Ferrigno, Edgar Díaz-Hernández, Elizabeth N. Holly, David J. Margolis, Eugenio Piasini, Marc V. Fuccillo

## Abstract

The dorsomedial striatum guides learning and adaptive decision-making through excitatory synaptic control of its spiny projection neuron outputs. However, the contributions of local inhibitory microcircuitry remain poorly understood. Here, we identify an interneuron circuit in the dorsomedial striatum that links outcome processing to adaptive action selection. During probabilistic push-pull reversal learning, interneurons represented immediate outcomes: somatostatin interneurons were recruited on unrewarded trials and unexpected rewarded trials, whereas tyrosine hydroxylase interneurons were suppressed on unrewarded trials and recruited on rewarded trials. In vivo recruitment of tyrosine hydroxylase interneurons suppressed somatostatin interneuron activity and increased activity in both direct- and indirect-pathway striatal projection neurons, revealing a polysynaptic disinhibitory microcircuit. Transient inhibition of somatostatin interneurons in this pull-tuned region produced a sustained increase in aberrant pull choices and occupancy of a suboptimal pull-preferring behavioral state, whereas inhibition of tyrosine hydroxylase interneurons produced a sustained impairment of pull reinforcement. Longer-term policy changes following somatostatin interneuron inhibition coincided with postsynaptic potentiation of excitatory synapses onto striatal projection neurons, suggesting a potential substrate for the persistence of altered behavioral policies. Together, these findings identify a disinhibitory striatal circuit motif gating reinforcement which transforms individual trial outcomes into temporally broader policy.

**Highlights:**

- Local striatal interneurons transform outcome signals into action-specific reinforcement.
- Tyrosine hydroxylase interneurons disinhibit striatal projection neurons through somatostatin interneurons.
- Transient interneuron inhibition drives persistent, opponent shifts in action policy.
- Somatostatin interneuron inhibition couples altered policy to excitatory synaptic potentiation.

## Full Text Availability

The license terms selected by the author(s) for this preprint version do not permit archiving in PMC. The full text is available from the preprint server.

